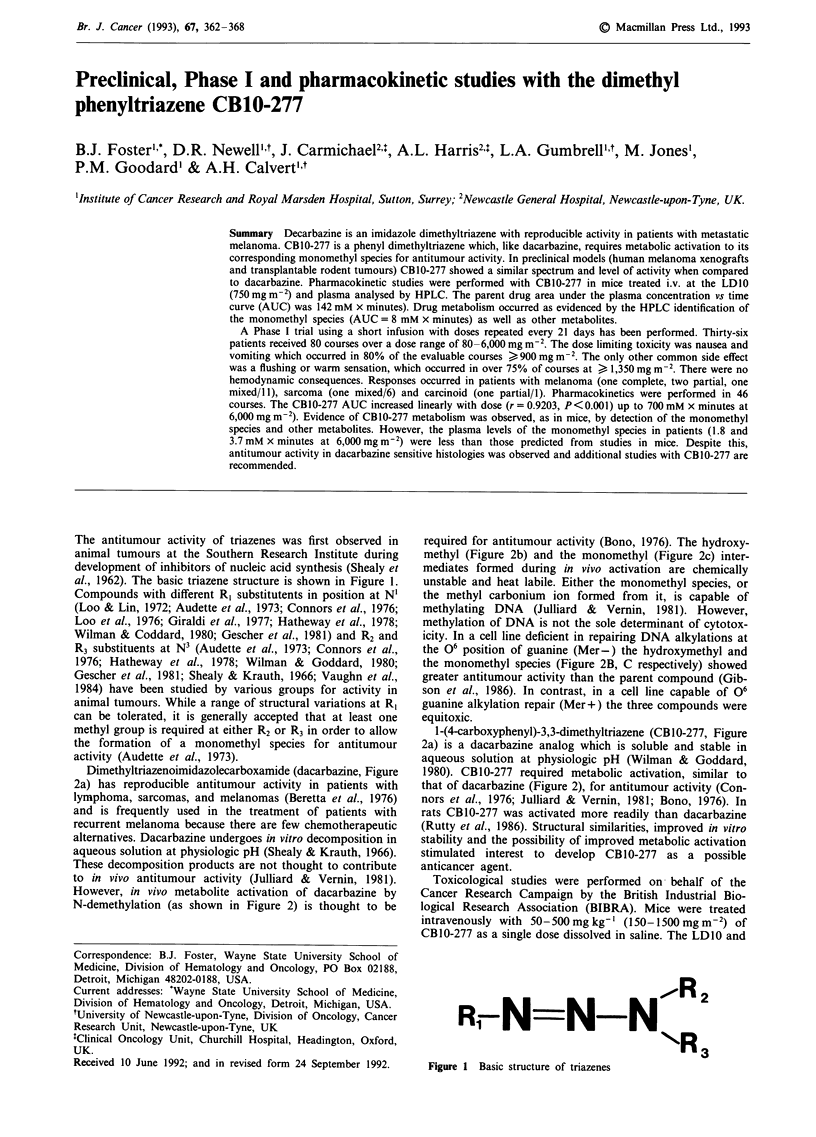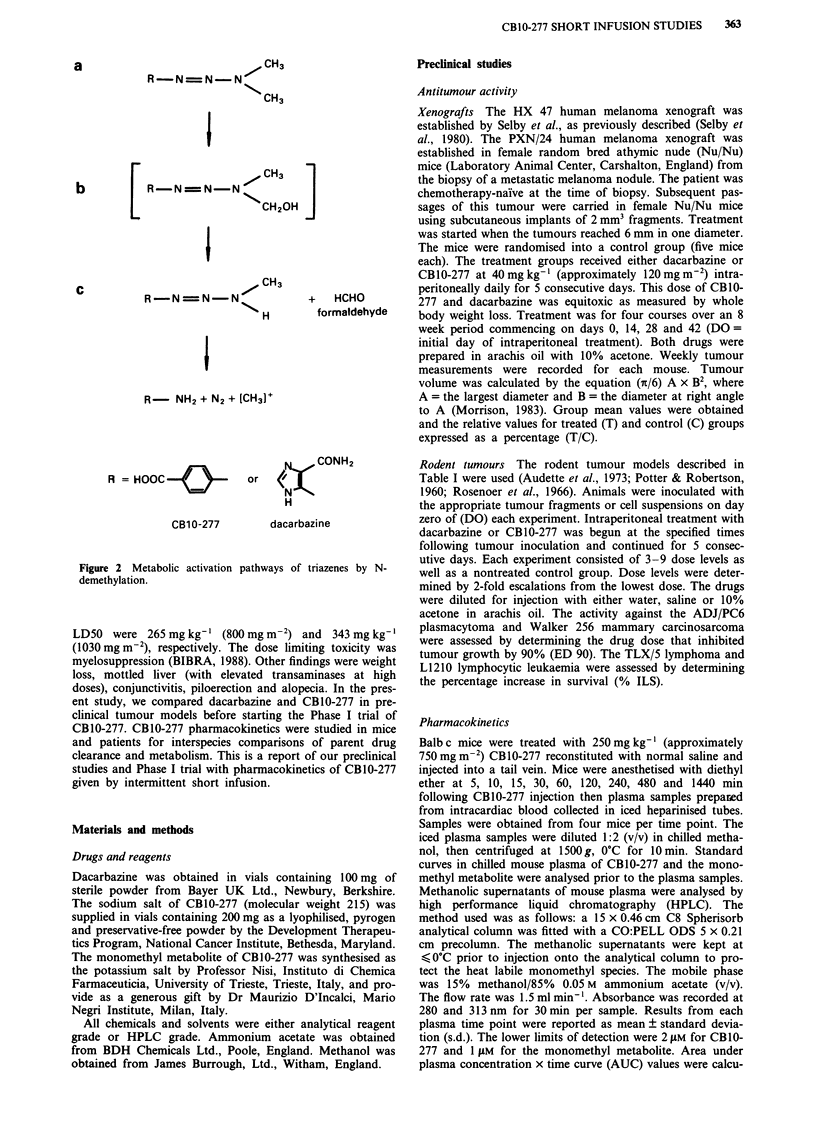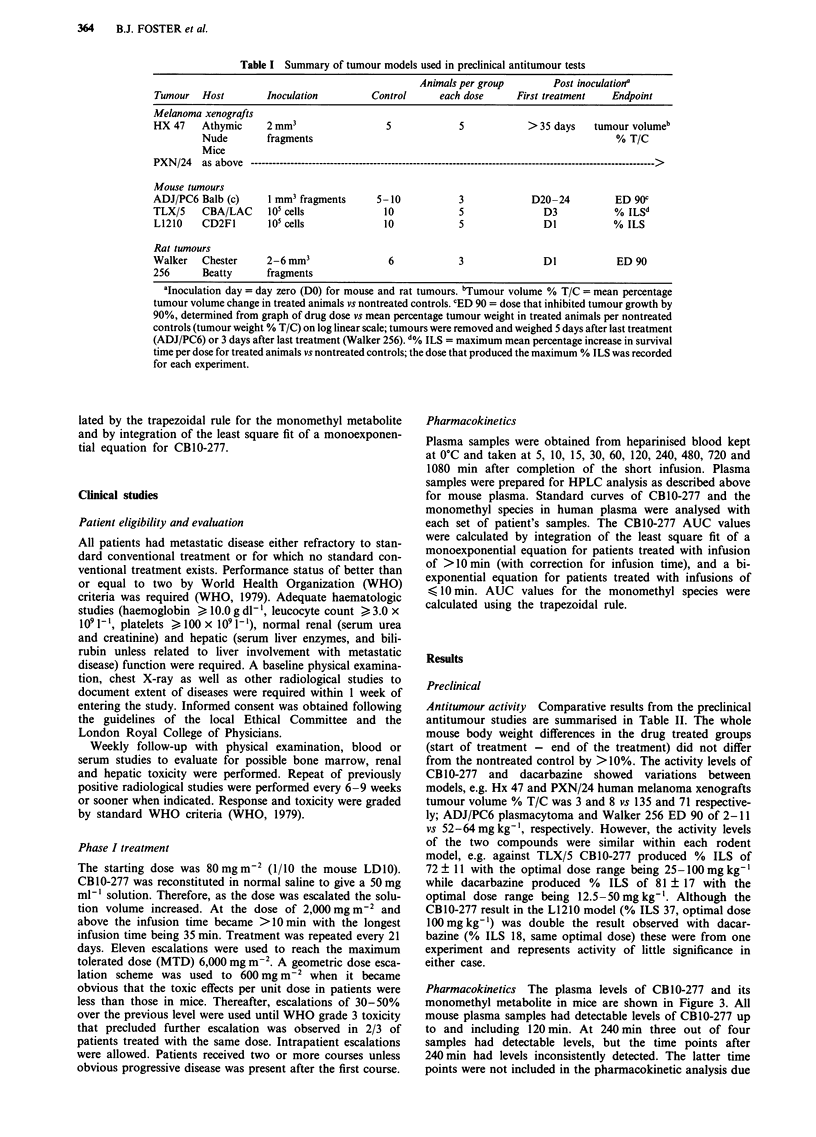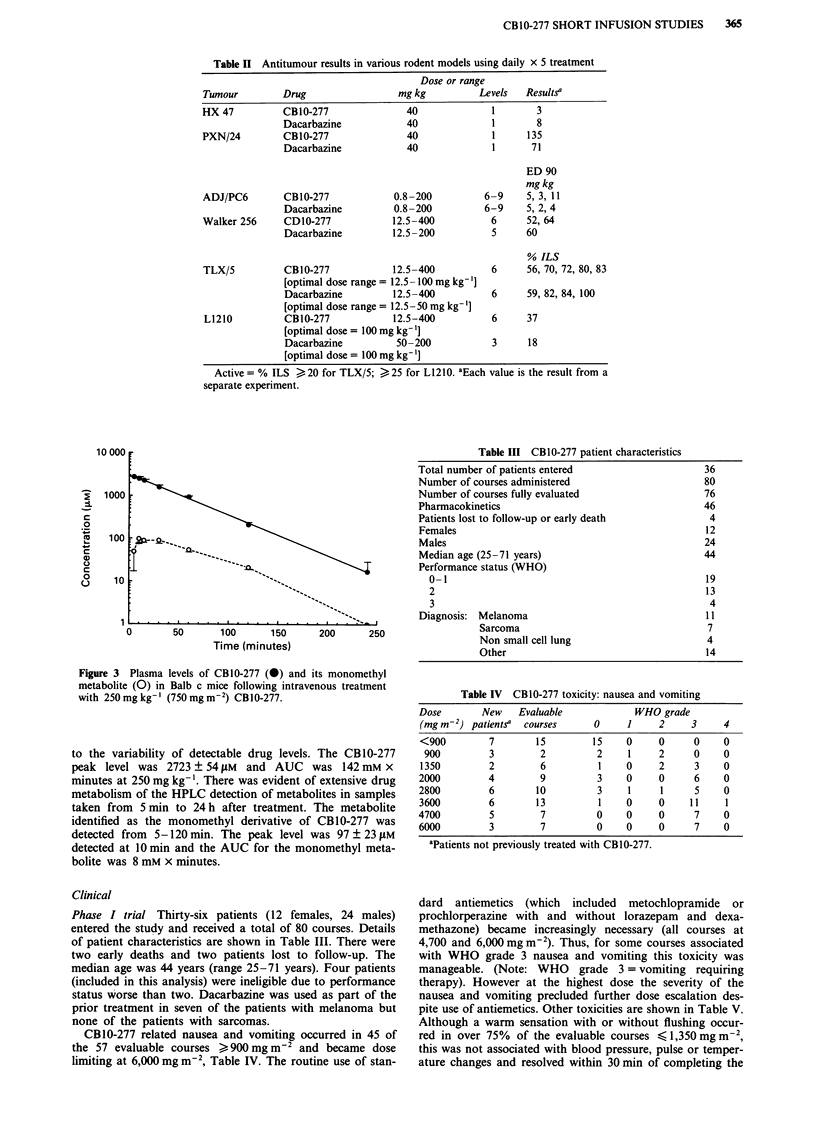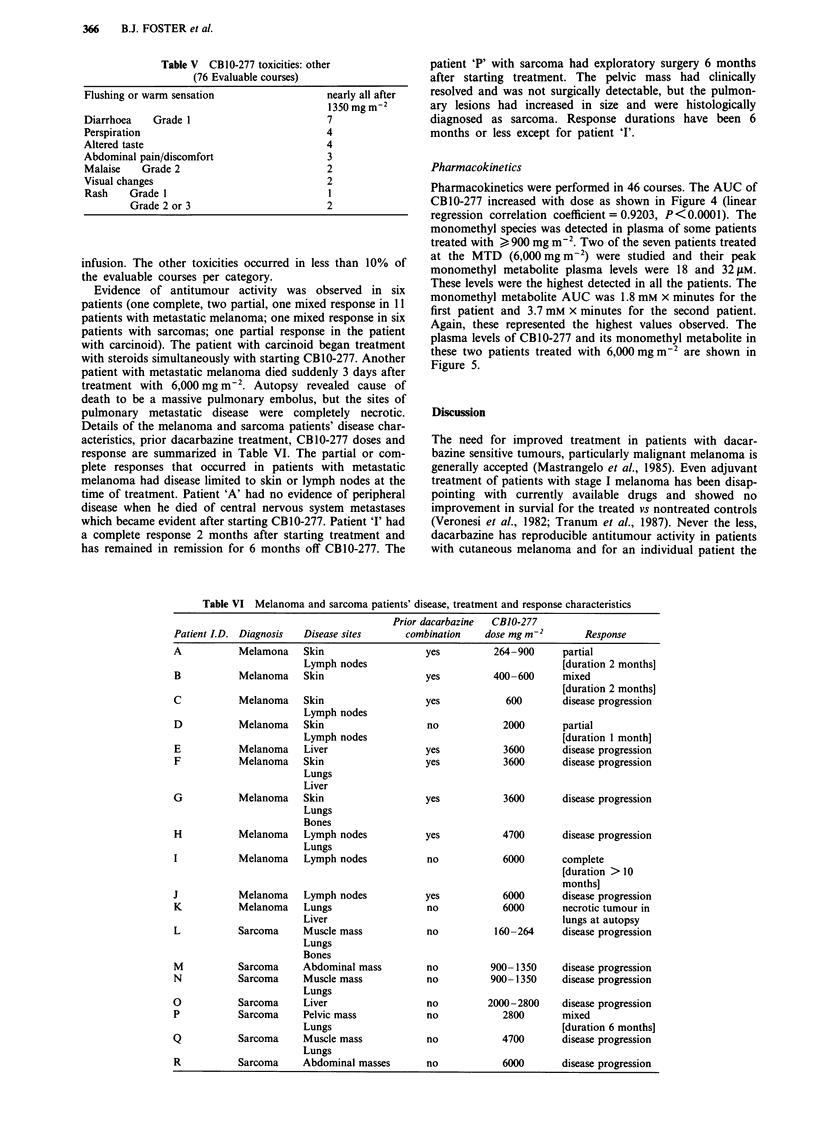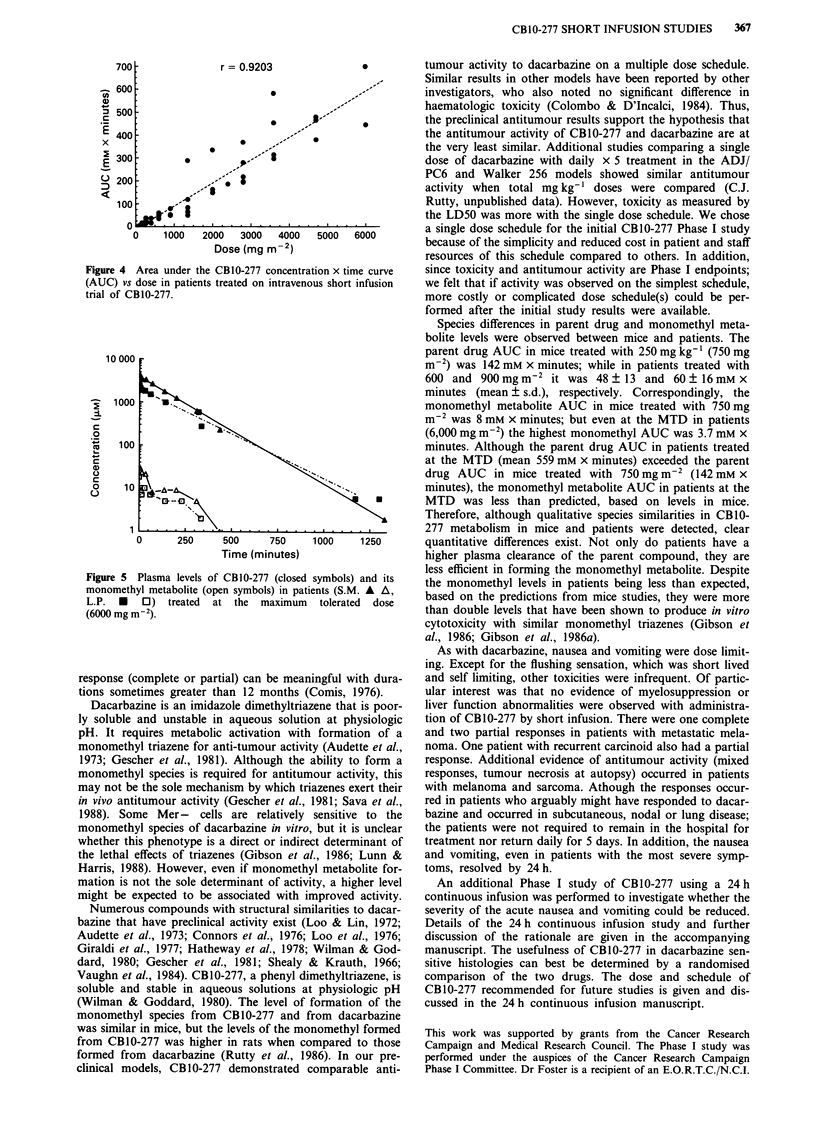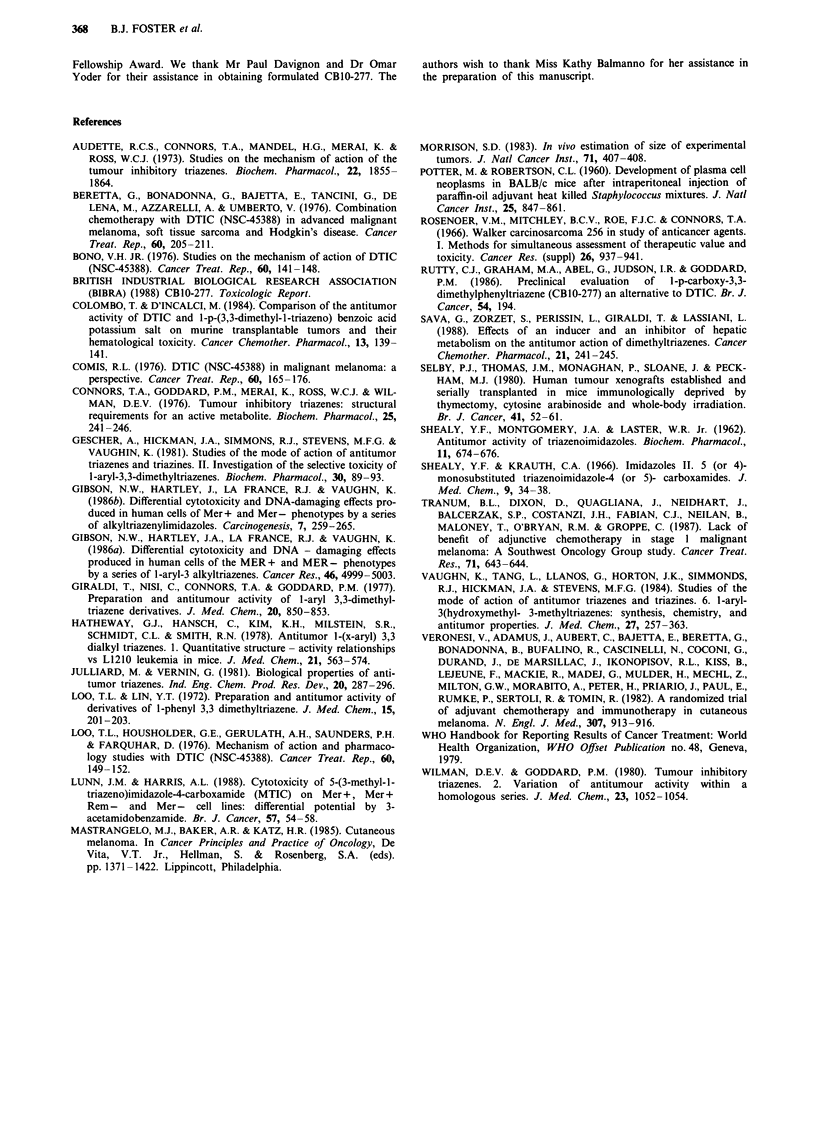# Preclinical, phase I and pharmacokinetic studies with the dimethyl phenyltriazene CB10-277.

**DOI:** 10.1038/bjc.1993.66

**Published:** 1993-02

**Authors:** B. J. Foster, D. R. Newell, J. Carmichael, A. L. Harris, L. A. Gumbrell, M. Jones, P. M. Goodard, A. H. Calvert

**Affiliations:** Institute of Cancer Research, Sutton, Surrey, UK.

## Abstract

Decarbazine is an imidazole dimethyltriazene with reproducible activity in patients with metastatic melanoma. CB10-277 is a phenyl dimethyltriazene which, like dacarbazine, requires metabolic activation to its corresponding monomethyl species for antitumour activity. In preclinical models (human melanoma xenografts and transplantable rodent tumours) CB10-277 showed a similar spectrum and level of activity when compared to dacarbazine. Pharmacokinetic studies were performed with CB10-277 in mice treated i.v. at the LD10 (750 mg m-2) and plasma analysed by HPLC. The parent drug area under the plasma concentration vs time curve (AUC) was 142 mM x minutes. Drug metabolism occurred as evidenced by the HPLC identification of the monomethyl species (AUC = 8 mM x minutes) as well as other metabolites. A Phase I trial using a short infusion with doses repeated every 21 days has been performed. Thirty-six patients received 80 courses over a dose range of 80-6,000 mg m-2. The dose limiting toxicity was nausea and vomiting which occurred in 80% of the evaluable courses > or = 900 mg m-2. The only other common side effect was a flushing or warm sensation, which occurred in over 75% of courses at > or = 1,350 mg m-2. There were no hemodynamic consequences. Responses occurred in patients with melanoma (one complete, two partial, one mixed/11), sarcoma (one mixed/6) and carcinoid (one partial/l). Pharmacokinetics were performed in 46 courses. The CB10-277 AUC increased linearly with dose (r = 0.9203, P < 0.001) up to 700 mM x minutes at 6,000 mg m-2). Evidence of CB10-277 metabolism was observed, as in mice, by detection of the monomethyl species and other metabolites. However, the plasma levels of the monomethyl species in patients (1.8 and 3.7 mM x minutes at 6,000 mg m-2) were less than those predicted from studies in mice. Despite this, antitumour activity in dacarbazine sensitive histologies was observed and additional studies with CB10-277 are recommended.


*Br. J. Cancer* (1993), **67**, 362–368 © Macmillan Press Ltd., 1993

### Preclinical, Phase I and pharmacokinetic studies with the dimethyl phenyltriazene CB10-277

B.J. Foster[1,*], D.R. Newell[1,†], J. Carmichael[2,‡], A.L. Harris[2,‡], L.A. Gumbrell[1,†], M. Jones[1], P.M. Goodard[1] & A.H. Calvert[1,†]

[1]*Institute of Cancer Research and Royal Marsden Hospital, Sutton, Surrey;* [2]*Newcastle General Hospital, Newcastle-upon-Tyne, UK.*

**Summary** Decarbazine is an imidazole dimethyltriazene with reproducible activity in patients with metastatic melanoma. CB10-277 is a phenyl dimethyltriazene which, like dacarbazine, requires metabolic activation to its corresponding monomethyl species for antitumour activity. In preclinical models (human melanoma xenografts and transplantable rodent tumours) CB10-277 showed a similar spectrum and level of activity when compared to dacarbazine. Pharmacokinetic studies were performed with CB10-277 in mice treated i.v. at the LD10 (750 mg m$^{-2}$) and plasma analysed by HPLC. The parent drug area under the plasma concentration *vs* time curve (AUC) was 142 mM × minutes). Drug metabolism occurred as evidenced by the HPLC identification of the monomethyl species (AUC = 8 mM × minutes) as well as other metabolites.

A Phase I trial using a short infusion with doses repeated every 21 days has been performed. Thirty-six patients received 80 courses over a dose range of 80–6,000 mg m$^{-2}$. The dose limiting toxicity was nausea and vomiting which occurred in 80% of the evaluable courses $\geqslant$900 mg m$^{-2}$. The only other common side effect was a flushing or warm sensation, which occurred in over 75% of courses at $\geqslant$1,350 mg m$^{-2}$. There were no hemodynamic consequences. Responses occurred in patients with melanoma (one complete, two partial, one mixed/11), sarcoma (one mixed/6) and carcinoid (one partial/1). Pharmacokinetics were performed in 46 courses. The CB10-277 AUC increased linearly with dose ($r$ = 0.9203, $P$<0.001) up to 700 mM × minutes at 6,000 mg m$^{-2}$). Evidence of CB10-277 metabolism was observed, as in mice, by detection of the monomethyl species and other metabolites. However, the plasma levels of the monomethyl species in patients (1.8 and 3.7 mM × minutes at 6,000 mg m$^{-2}$) were less than those predicted from studies in mice. Despite this, antitumour activity in dacarbazine sensitive histologies was observed and additional studies with CB10-277 are recommended.

The antitumour activity of triazenes was first observed in animal tumours at the Southern Research Institute during development of inhibitors of nucleic acid synthesis (Shealy *et al.*, 1962). The basic triazene structure is shown in Figure 1. Compounds with different $R_1$ substitutents in position at $N^1$ (Loo & Lin, 1972; Audette *et al.*, 1973; Connors *et al.*, 1976; Loo *et al.*, 1976; Giraldi *et al.*, 1977; Hatheway *et al.*, 1978; Wilman & Coddard, 1980; Gescher *et al.*, 1981) and $R_2$ and $R_3$ substituents at $N^3$ (Audette *et al.*, 1973; Connors *et al.*, 1976; Hatheway *et al.*, 1978; Wilman & Goddard, 1980; Gescher *et al.*, 1981; Shealy & Krauth, 1966; Vaughn *et al.*, 1984) have been studied by various groups for activity in animal tumours. While a range of structural variations at $R_1$ can be tolerated, it is generally accepted that at least one methyl group is required at either $R_2$ or $R_3$ in order to allow the formation of a monomethyl species for antitumour activity (Audette *et al.*, 1973).

Dimethyltriazenoimidazolecarboxamide (dacarbazine, Figure 2a) has reproducible antitumour activity in patients with lymphoma, sarcomas, and melanomas (Beretta *et al.*, 1976) and is frequently used in the treatment of patients with recurrent melanoma because there are few chemotherapeutic alternatives. Dacarbazine undergoes *in vitro* decomposition in aqueous solution at physiologic pH (Shealy & Krauth, 1966). These decomposition products are not thought to contribute to *in vivo* antitumour activity (Julliard & Vernin, 1981). However, *in vivo* metabolite activation of dacarbazine by N-demethylation (as shown in Figure 2) is thought to be required for antitumour activity (Bono, 1976). The hydroxymethyl (Figure 2b) and the monomethyl (Figure 2c) intermediates formed during *in vivo* activation are chemically unstable and heat labile. Either the monomethyl species, or the methyl carbonium ion formed from it, is capable of methylating DNA (Julliard & Vernin, 1981). However, methylation of DNA is not the sole determinant of cytotoxicity. In a cell line deficient in repairing DNA alkylations at the $O^6$ position of guanine (Mer−) the hydroxymethyl and the monomethyl species (Figure 2B, C respectively) showed greater antitumour activity than the parent compound (Gibson *et al.*, 1986). In contrast, in a cell line capable of $O^6$ guanine alkylation repair (Mer+) the three compounds were equitoxic.

1-(4-carboxyphenyl)-3,3-dimethyltriazene (CB10-277, Figure 2a) is a dacarbazine analog which is soluble and stable in aqueous solution at physiologic pH (Wilman & Goddard, 1980). CB10-277 required metabolic activation, similar to that of dacarbazine (Figure 2), for antitumour activity (Connors *et al.*, 1976; Julliard & Vernin, 1981; Bono, 1976). In rats CB10-277 was activated more readily than dacarbazine (Rutty *et al.*, 1986). Structural similarities, improved *in vitro* stability and the possibility of improved metabolic activation stimulated interest to develop CB10-277 as a possible anticancer agent.

Toxicological studies were performed on behalf of the Cancer Research Campaign by the British Industrial Biological Research Association (BIBRA). Mice were treated intravenously with 50–500 mg kg$^{-1}$ (150–1500 mg m$^{-2}$) of CB10-277 as a single dose dissolved in saline. The LD10 and

**Figure 1** Basic structure of triazenes

Correspondence: B.J. Foster, Wayne State University School of Medicine, Division of Hematology and Oncology, PO Box 02188, Detroit, Michigan 48202-0188, USA.
Current addresses: *Wayne State University School of Medicine, Division of Hematology and Oncology, Detroit, Michigan, USA. †University of Newcastle-upon-Tyne, Division of Oncology, Cancer Research Unit, Newcastle-upon-Tyne, UK
‡Clinical Oncology Unit, Churchill Hospital, Headington, Oxford, UK.
Received 10 June 1992; and in revised form 24 September 1992.

**Figure 2** Metabolic activation pathways of triazenes by N-demethylation.

LD50 were 265 mg kg$^{-1}$ (800 mg m$^{-2}$) and 343 mg kg$^{-1}$ (1030 mg m$^{-2}$), respectively. The dose limiting toxicity was myelosuppression (BIBRA, 1988). Other findings were weight loss, mottled liver (with elevated transaminases at high doses), conjunctivitis, piloerection and alopecia. In the present study, we compared dacarbazine and CB10-277 in preclinical tumour models before starting the Phase I trial of CB10-277. CB10-277 pharmacokinetics were studied in mice and patients for interspecies comparisons of parent drug clearance and metabolism. This is a report of our preclinical studies and Phase I trial with pharmacokinetics of CB10-277 given by intermittent short infusion.

### Materials and methods

### *Drugs and reagents*

Dacarbazine was obtained in vials containing 100 mg of sterile powder from Bayer UK Ltd., Newbury, Berkshire. The sodium salt of CB10-277 (molecular weight 215) was supplied in vials containing 200 mg as a lyophilised, pyrogen and preservative-free powder by the Development Therapeutics Program, National Cancer Institute, Bethesda, Maryland. The monomethyl metabolite of CB10-277 was synthesised as the potassium salt by Professor Nisi, Instituto di Chemica Farmaceuticia, University of Trieste, Trieste, Italy, and provide as a generous gift by Dr Maurizio D'Incalci, Mario Negri Institute, Milan, Italy.

All chemicals and solvents were either analytical reagent grade or HPLC grade. Ammonium acetate was obtained from BDH Chemicals Ltd., Poole, England. Methanol was obtained from James Burrough, Ltd., Witham, England.

### Preclinical studies

### *Antitumour activity*

*Xenografts* The HX 47 human melanoma xenograft was established by Selby *et al.*, as previously described (Selby *et al.*, 1980). The PXN/24 human melanoma xenograft was established in female random bred athymic nude (Nu/Nu) mice (Laboratory Animal Center, Carshalton, England) from the biopsy of a metastatic melanoma nodule. The patient was chemotherapy-naïve at the time of biopsy. Subsequent passages of this tumour were carried in female Nu/Nu mice using subcutaneous implants of 2 mm$^3$ fragments. Treatment was started when the tumours reached 6 mm in one diameter. The mice were randomised into a control group (five mice each). The treatment groups received either dacarbazine or CB10-277 at 40 mg kg$^{-1}$ (approximately 120 mg m$^{-2}$) intraperitoneally daily for 5 consecutive days. This dose of CB10-277 and dacarbazine was equitoxic as measured by whole body weight loss. Treatment was for four courses over an 8 week period commencing on days 0, 14, 28 and 42 (DO = initial day of intraperitoneal treatment). Both drugs were prepared in arachis oil with 10% acetone. Weekly tumour measurements were recorded for each mouse. Tumour volume was calculated by the equation ($\pi$/6) A $\times$ B$^2$, where A = the largest diameter and B = the diameter at right angle to A (Morrison, 1983). Group mean values were obtained and the relative values for treated (T) and control (C) groups expressed as a percentage (T/C).

*Rodent tumours* The rodent tumour models described in Table I were used (Audette *et al.*, 1973; Potter & Robertson, 1960; Rosenoer *et al.*, 1966). Animals were inoculated with the appropriate tumour fragments or cell suspensions on day zero of (DO) each experiment. Intraperitoneal treatment with dacarbazine or CB10-277 was begun at the specified times following tumour inoculation and continued for 5 consecutive days. Each experiment consisted of 3–9 dose levels as well as a nontreated control group. Dose levels were determined by 2-fold escalations from the lowest dose. The drugs were diluted for injection with either water, saline or 10% acetone in arachis oil. The activity against the ADJ/PC6 plasmacytoma and Walker 256 mammary carcinosarcoma were assessed by determining the drug dose that inhibited tumour growth by 90% (ED 90). The TLX/5 lymphoma and L1210 lymphocytic leukaemia were assessed by determining the percentage increase in survival (% ILS).

### *Pharmacokinetics*

Balb c mice were treated with 250 mg kg$^{-1}$ (approximately 750 mg m$^{-2}$) CB10-277 reconstituted with normal saline and injected into a tail vein. Mice were anesthetised with diethyl ether at 5, 10, 15, 30, 60, 120, 240, 480 and 1440 min following CB10-277 injection then plasma samples prepared from intracardiac blood collected in iced heparinised tubes. Samples were obtained from four mice per time point. The iced plasma samples were diluted 1:2 (v/v) in chilled methanol, then centrifuged at 1500 *g*, 0°C for 10 min. Standard curves in chilled mouse plasma of CB10-277 and the monomethyl metabolite were analysed prior to the plasma samples. Methanolic supernatants of mouse plasma were analysed by high performance liquid chromatography (HPLC). The method used was as follows: a 15 $\times$ 0.46 cm C8 Spherisorb analytical column was fitted with a CO:PELL ODS 5 $\times$ 0.21 cm precolumn. The methanolic supernatants were kept at $\leqslant$0°C prior to injection onto the analytical column to protect the heat labile monomethyl species. The mobile phase was 15% methanol/85% 0.05 M ammonium acetate (v/v). The flow rate was 1.5 ml min$^{-1}$. Absorbance was recorded at 280 and 313 nm for 30 min per sample. Results from each plasma time point were reported as mean $\pm$ standard deviation (s.d.). The lower limits of detection were 2 $\mu$M for CB10-277 and 1 $\mu$M for the monomethyl metabolite. Area under plasma concentration $\times$ time curve (AUC) values were calcu-

**Table I** Summary of tumour models used in preclinical antitumour tests

| Tumour | Host | Inoculation | Control | Animals per group each dose | Post inoculation[a] First treatment | Endpoint |
|---|---|---|---|---|---|---|
| *Melanoma xenografts* | | | | | | |
| HX 47 | Athymic Nude Mice | 2 mm³ fragments | 5 | 5 | >35 days | tumour volume[b] % T/C |
| PXN/24 | as above | ---------------------------------------------------------> | | | | |
| *Mouse tumours* | | | | | | |
| ADJ/PC6 | Balb (c) | 1 mm³ fragments | 5–10 | 3 | D20–24 | ED 90[c] |
| TLX/5 | CBA/LAC | $10^5$ cells | 10 | 5 | D3 | % ILS[d] |
| L1210 | CD2F1 | $10^5$ cells | 10 | 5 | D1 | % ILS |
| *Rat tumours* | | | | | | |
| Walker 256 | Chester Beatty | 2–6 mm³ fragments | 6 | 3 | D1 | ED 90 |

[a]Inoculation day = day zero (D0) for mouse and rat tumours. [b]Tumour volume % T/C = mean percentage tumour volume change in treated animals *vs* nontreated controls. [c]ED 90 = dose that inhibited tumour growth by 90%, determined from graph of drug dose *vs* mean percentage tumour weight in treated animals per nontreated controls (tumour weight % T/C) on log linear scale; tumours were removed and weighed 5 days after last treatment (ADJ/PC6) or 3 days after last treatment (Walker 256). [d]% ILS = maximum mean percentage increase in survival time per dose for treated animals *vs* nontreated controls; the dose that produced the maximum % ILS was recorded for each experiment.

lated by the trapezoidal rule for the monomethyl metabolite and by integration of the least square fit of a monoexponential equation for CB10-277.

### Clinical studies

*Patient eligibility and evaluation*

All patients had metastatic disease either refractory to standard conventional treatment or for which no standard conventional treatment exists. Performance status of better than or equal to two by World Health Organization (WHO) criteria was required (WHO, 1979). Adequate haematologic studies (haemoglobin $\geqslant 10.0\ g\ dl^{-1}$, leucocyte count $\geqslant 3.0 \times 10^9\ l^{-1}$, platelets $\geqslant 100 \times 10^9\ l^{-1}$), normal renal (serum urea and creatinine) and hepatic (serum liver enzymes, and bilirubin unless related to liver involvement with metastatic disease) function were required. A baseline physical examination, chest X-ray as well as other radiological studies to document extent of diseases were required within 1 week of entering the study. Informed consent was obtained following the guidelines of the local Ethical Committee and the London Royal College of Physicians.

Weekly follow-up with physical examination, blood or serum studies to evaluate for possible bone marrow, renal and hepatic toxicity were performed. Repeat of previously positive radiological studies were performed every 6–9 weeks or sooner when indicated. Response and toxicity were graded by standard WHO criteria (WHO, 1979).

*Phase I treatment*

The starting dose was $80\ mg\ m^{-2}$ (1/10 the mouse LD10). CB10-277 was reconstituted in normal saline to give a 50 mg $ml^{-1}$ solution. Therefore, as the dose was escalated the solution volume increased. At the dose of $2{,}000\ mg\ m^{-2}$ and above the infusion time became >10 min with the longest infusion time being 35 min. Treatment was repeated every 21 days. Eleven escalations were used to reach the maximum tolerated dose (MTD) $6{,}000\ mg\ m^{-2}$. A geometric dose escalation scheme was used to $600\ mg\ m^{-2}$ when it became obvious that the toxic effects per unit dose in patients were less than those in mice. Thereafter, escalations of 30–50% over the previous level were used until WHO grade 3 toxicity that precluded further escalation was observed in 2/3 of patients treated with the same dose. Intrapatient escalations were allowed. Patients received two or more courses unless obvious progressive disease was present after the first course.

*Pharmacokinetics*

Plasma samples were obtained from heparinised blood kept at 0°C and taken at 5, 10, 15, 30, 60, 120, 240, 480, 720 and 1080 min after completion of the short infusion. Plasma samples were prepared for HPLC analysis as described above for mouse plasma. Standard curves of CB10-277 and the monomethyl species in human plasma were analysed with each set of patient's samples. The CB10-277 AUC values were calculated by integration of the least square fit of a monoexponential equation for patients treated with infusion of >10 min (with correction for infusion time), and a biexponential equation for patients treated with infusions of $\leqslant 10$ min. AUC values for the monomethyl species were calculated using the trapezoidal rule.

### Results

*Preclinical*

*Antitumour activity* Comparative results from the preclinical antitumour studies are summarised in Table II. The whole mouse body weight differences in the drug treated groups (start of treatment – end of the treatment) did not differ from the nontreated control by >10%. The activity levels of CB10-277 and dacarbazine showed variations between models, e.g. Hx 47 and PXN/24 human melanoma xenografts tumour volume % T/C was 3 and 8 *vs* 135 and 71 respectively; ADJ/PC6 plasmacytoma and Walker 256 ED 90 of 2–11 *vs* $52–64\ mg\ kg^{-1}$, respectively. However, the activity levels of the two compounds were similar within each rodent model, e.g. against TLX/5 CB10-277 produced % ILS of $72 \pm 11$ with the optimal dose range being $25–100\ mg\ kg^{-1}$ while dacarbazine produced % ILS of $81 \pm 17$ with the optimal dose range being $12.5–50\ mg\ kg^{-1}$. Although the CB10-277 result in the L1210 model (% ILS 37, optimal dose $100\ mg\ kg^{-1}$) was double the result observed with dacarbazine (% ILS 18, same optimal dose) these were from one experiment and represents activity of little significance in either case.

*Pharmacokinetics* The plasma levels of CB10-277 and its monomethyl metabolite in mice are shown in Figure 3. All mouse plasma samples had detectable levels of CB10-277 up to and including 120 min. At 240 min three out of four samples had detectable levels, but the time points after 240 min had levels inconsistently detected. The latter time points were not included in the pharmacokinetic analysis due

**Table II** Antitumour results in various rodent models using daily × 5 treatment

| *Tumour* | *Drug* | *Dose or range* *mg kg* | *Levels* | *Results*[a] |
|---|---|---|---|---|
| HX 47 | CB10-277 | 40 | 1 | 3 |
| | Dacarbazine | 40 | 1 | 8 |
| PXN/24 | CB10-277 | 40 | 1 | 135 |
| | Dacarbazine | 40 | 1 | 71 |
| | | | | ED 90 *mg kg* |
| ADJ/PC6 | CB10-277 | 0.8–200 | 6–9 | 5, 3, 11 |
| | Dacarbazine | 0.8–200 | 6–9 | 5, 2, 4 |
| Walker 256 | CD10-277 | 12.5–400 | 6 | 52, 64 |
| | Dacarbazine | 12.5–200 | 5 | 60 |
| | | | | *% ILS* |
| TLX/5 | CB10-277 | 12.5–400 | 6 | 56, 70, 72, 80, 83 |
| | [optimal dose range = 12.5–100 mg kg$^{-1}$] | | | |
| | Dacarbazine | 12.5–400 | 6 | 59, 82, 84, 100 |
| | [optimal dose range = 12.5–50 mg kg$^{-1}$] | | | |
| L1210 | CB10-277 | 12.5–400 | 6 | 37 |
| | [optimal dose = 100 mg kg$^{-1}$] | | | |
| | Dacarbazine | 50–200 | 3 | 18 |
| | [optimal dose = 100 mg kg$^{-1}$] | | | |

Active = % ILS ⩾20 for TLX/5; ⩾25 for L1210. [a]Each value is the result from a separate experiment.

**Figure 3** Plasma levels of CB10-277 (●) and its monomethyl metabolite (○) in Balb c mice following intravenous treatment with 250 mg kg$^{-1}$ (750 mg m$^{-2}$) CB10-277.

to the variability of detectable drug levels. The CB10-277 peak level was 2723 ± 54 µM and AUC was 142 mM × minutes at 250 mg kg$^{-1}$. There was evident of extensive drug metabolism of the HPLC detection of metabolites in samples taken from 5 min to 24 h after treatment. The metabolite identified as the monomethyl derivative of CB10-277 was detected from 5–120 min. The peak level was 97 ± 23 µM detected at 10 min and the AUC for the monomethyl metabolite was 8 mM × minutes.

**Table III** CB10-277 patient characteristics

| | |
|---|---|
| Total number of patients entered | 36 |
| Number of courses administered | 80 |
| Number of courses fully evaluated | 76 |
| Pharmacokinetics | 46 |
| Patients lost to follow-up or early death | 4 |
| Females | 12 |
| Males | 24 |
| Median age (25–71 years) | 44 |
| Performance status (WHO) | |
| 0–1 | 19 |
| 2 | 13 |
| 3 | 4 |
| Diagnosis: Melanoma | 11 |
| Sarcoma | 7 |
| Non small cell lung | 4 |
| Other | 14 |

**Table IV** CB10-277 toxicity: nausea and vomiting

| *Dose (mg m$^{-2}$)* | *New patients*[a] | *Evaluable courses* | *WHO grade* 0 | 1 | 2 | 3 | 4 |
|---|---|---|---|---|---|---|---|
| <900 | 7 | 15 | 15 | 0 | 0 | 0 | 0 |
| 900 | 3 | 2 | 2 | 1 | 2 | 0 | 0 |
| 1350 | 2 | 6 | 1 | 0 | 2 | 3 | 0 |
| 2000 | 4 | 9 | 3 | 0 | 0 | 6 | 0 |
| 2800 | 6 | 10 | 3 | 1 | 1 | 5 | 0 |
| 3600 | 6 | 13 | 1 | 0 | 0 | 11 | 1 |
| 4700 | 5 | 7 | 0 | 0 | 0 | 7 | 0 |
| 6000 | 3 | 7 | 0 | 0 | 0 | 7 | 0 |

[a]Patients not previously treated with CB10-277.

### *Clinical*

*Phase I trial* Thirty-six patients (12 females, 24 males) entered the study and received a total of 80 courses. Details of patient characteristics are shown in Table III. There were two early deaths and two patients lost to follow-up. The median age was 44 years (range 25–71 years). Four patients (included in this analysis) were ineligible due to performance status worse than two. Dacarbazine was used as part of the prior treatment in seven of the patients with melanoma but none of the patients with sarcomas.

CB10-277 related nausea and vomiting occurred in 45 of the 57 evaluable courses ⩾900 mg m$^{-2}$ and became dose limiting at 6,000 mg m$^{-2}$, Table IV. The routine use of standard antiemetics (which included metochlopramide or prochlorperazine with and without lorazepam and dexamethazone) became increasingly necessary (all courses at 4,700 and 6,000 mg m$^{-2}$). Thus, for some courses associated with WHO grade 3 nausea and vomiting this toxicity was manageable. (Note: WHO grade 3 = vomiting requiring therapy). However at the highest dose the severity of the nausea and vomiting precluded further dose escalation despite use of antiemetics. Other toxicities are shown in Table V. Although a warm sensation with or without flushing occurred in over 75% of the evaluable courses ⩽1,350 mg m$^{-2}$, this was not associated with blood pressure, pulse or temperature changes and resolved within 30 min of completing the

**Table V** CB10-277 toxicities: other (76 Evaluable courses)

| | | |
|---|---|---|
| Flushing or warm sensation | | nearly all after 1350 mg m$^{-2}$ |
| Diarrhoea | Grade 1 | 7 |
| Perspiration | | 4 |
| Altered taste | | 4 |
| Abdominal pain/discomfort | | 3 |
| Malaise | Grade 2 | 2 |
| Visual changes | | 2 |
| Rash | Grade 1 | 1 |
| | Grade 2 or 3 | 2 |

infusion. The other toxicities occurred in less than 10% of the evaluable courses per category.

Evidence of antitumour activity was observed in six patients (one complete, two partial, one mixed response in 11 patients with metastatic melanoma; one mixed response in six patients with sarcomas; one partial response in the patient with carcinoid). The patient with carcinoid began treatment with steroids simultaneously with starting CB10-277. Another patient with metastatic melanoma died suddenly 3 days after treatment with 6,000 mg m$^{-2}$. Autopsy revealed cause of death to be a massive pulmonary embolus, but the sites of pulmonary metastatic disease were completely necrotic. Details of the melanoma and sarcoma patients' disease characteristics, prior dacarbazine treatment, CB10-277 doses and response are summarized in Table VI. The partial or complete responses that occurred in patients with metastatic melanoma had disease limited to skin or lymph nodes at the time of treatment. Patient 'A' had no evidence of peripheral disease when he died of central nervous system metastases which became evident after starting CB10-277. Patient 'I' had a complete response 2 months after starting treatment and has remained in remission for 6 months off CB10-277. The patient 'P' with sarcoma had exploratory surgery 6 months after starting treatment. The pelvic mass had clinically resolved and was not surgically detectable, but the pulmonary lesions had increased in size and were histologically diagnosed as sarcoma. Response durations have been 6 months or less except for patient 'I'.

*Pharmacokinetics*

Pharmacokinetics were performed in 46 courses. The AUC of CB10-277 increased with dose as shown in Figure 4 (linear regression correlation coefficient = 0.9203, $P<0.0001$). The monomethyl species was detected in plasma of some patients treated with $\geqslant$900 mg m$^{-2}$. Two of the seven patients treated at the MTD (6,000 mg m$^{-2}$) were studied and their peak monomethyl metabolite plasma levels were 18 and 32 µM. These levels were the highest detected in all the patients. The monomethyl metabolite AUC was 1.8 mM × minutes for the first patient and 3.7 mM × minutes for the second patient. Again, these represented the highest values observed. The plasma levels of CB10-277 and its monomethyl metabolite in these two patients treated with 6,000 mg m$^{-2}$ are shown in Figure 5.

### Discussion

The need for improved treatment in patients with dacarbazine sensitive tumours, particularly malignant melanoma is generally accepted (Mastrangelo *et al.*, 1985). Even adjuvant treatment of patients with stage I melanoma has been disappointing with currently available drugs and showed no improvement in survial for the treated *vs* nontreated controls (Veronesi *et al.*, 1982; Tranum *et al.*, 1987). Never the less, dacarbazine has reproducible antitumour activity in patients with cutaneous melanoma and for an individual patient the

**Table VI** Melanoma and sarcoma patients' disease, treatment and response characteristics

| *Patient I.D.* | *Diagnosis* | *Disease sites* | *Prior dacarbazine combination* | *CB10-277 dose mg m$^{-2}$* | *Response* |
|---|---|---|---|---|---|
| A | Melamona | Skin<br>Lymph nodes | yes | 264–900 | partial<br>[duration 2 months] |
| B | Melanoma | Skin | yes | 400–600 | mixed<br>[duration 2 months] |
| C | Melanoma | Skin<br>Lymph nodes | yes | 600 | disease progression |
| D | Melanoma | Skin<br>Lymph nodes | no | 2000 | partial<br>[duration 1 month] |
| E | Melanoma | Liver | yes | 3600 | disease progression |
| F | Melanoma | Skin<br>Lungs<br>Liver | yes | 3600 | disease progression |
| G | Melanoma | Skin<br>Lungs<br>Bones | yes | 3600 | disease progression |
| H | Melanoma | Lymph nodes<br>Lungs | yes | 4700 | disease progression |
| I | Melanoma | Lymph nodes | no | 6000 | complete<br>[duration >10 months] |
| J | Melanoma | Lymph nodes | yes | 6000 | disease progression |
| K | Melanoma | Lungs<br>Liver | no | 6000 | necrotic tumour in lungs at autopsy |
| L | Sarcoma | Muscle mass<br>Lungs<br>Bones | no | 160–264 | disease progression |
| M | Sarcoma | Abdominal mass | no | 900–1350 | disease progression |
| N | Sarcoma | Muscle mass<br>Lungs | no | 900–1350 | disease progression |
| O | Sarcoma | Liver | no | 2000–2800 | disease progression |
| P | Sarcoma | Pelvic mass<br>Lungs | no | 2800 | mixed<br>[duration 6 months] |
| Q | Sarcoma | Muscle mass<br>Lungs | no | 4700 | disease progression |
| R | Sarcoma | Abdominal masses | no | 6000 | disease progression |

**Figure 4** Area under the CB10-277 concentration × time curve (AUC) *vs* dose in patients treated on intravenous short infusion trial of CB10-277.

**Figure 5** Plasma levels of CB10-277 (closed symbols) and its monomethyl metabolite (open symbols) in patients (S.M. ▲ △, L.P. ■ □) treated at the maximum tolerated dose (6000 mg m$^{-2}$).

response (complete or partial) can be meaningful with durations sometimes greater than 12 months (Comis, 1976).

Dacarbazine is an imidazole dimethyltriazene that is poorly soluble and unstable in aqueous solution at physiologic pH. It requires metabolic activation with formation of a monomethyl triazene for anti-tumour activity (Audette *et al.*, 1973; Gescher *et al.*, 1981). Although the ability to form a monomethyl species is required for antitumour activity, this may not be the sole mechanism by which triazenes exert their *in vivo* antitumour activity (Gescher *et al.*, 1981; Sava *et al.*, 1988). Some Mer− cells are relatively sensitive to the monomethyl species of dacarbazine *in vitro*, but it is unclear whether this phenotype is a direct or indirect determinant of the lethal effects of triazenes (Gibson *et al.*, 1986; Lunn & Harris, 1988). However, even if monomethyl metabolite formation is not the sole determinant of activity, a higher level might be expected to be associated with improved activity.

Numerous compounds with structural similarities to dacarbazine that have preclinical activity exist (Loo & Lin, 1972; Audette *et al.*, 1973; Connors *et al.*, 1976; Loo *et al.*, 1976; Giraldi *et al.*, 1977; Hatheway *et al.*, 1978; Wilman & Goddard, 1980; Gescher *et al.*, 1981; Shealy & Krauth, 1966; Vaughn *et al.*, 1984). CB10-277, a phenyl dimethyltriazene, is soluble and stable in aqueous solutions at physiologic pH (Wilman & Goddard, 1980). The level of formation of the monomethyl species from CB10-277 and from dacarbazine was similar in mice, but the levels of the monomethyl formed from CB10-277 was higher in rats when compared to those formed from dacarbazine (Rutty *et al.*, 1986). In our preclinical models, CB10-277 demonstrated comparable antitumour activity to dacarbazine on a multiple dose schedule. Similar results in other models have been reported by other investigators, who also noted no significant difference in haematologic toxicity (Colombo & D'Incalci, 1984). Thus, the preclinical antitumour results support the hypothesis that the antitumour activity of CB10-277 and dacarbazine are at the very least similar. Additional studies comparing a single dose of dacarbazine with daily × 5 treatment in the ADJ/PC6 and Walker 256 models showed similar antitumour activity when total mg kg$^{-1}$ doses were compared (C.J. Rutty, unpublished data). However, toxicity as measured by the LD50 was more with the single dose schedule. We chose a single dose schedule for the initial CB10-277 Phase I study because of the simplicity and reduced cost in patient and staff resources of this schedule compared to others. In addition, since toxicity and antitumour activity are Phase I endpoints; we felt that if activity was observed on the simplest schedule, more costly or complicated dose schedule(s) could be performed after the initial study results were available.

Species differences in parent drug and monomethyl metabolite levels were observed between mice and patients. The parent drug AUC in mice treated with 250 mg kg$^{-1}$ (750 mg m$^{-2}$) was 142 mM × minutes; while in patients treated with 600 and 900 mg m$^{-2}$ it was 48 ± 13 and 60 ± 16 mM × minutes (mean ± s.d.), respectively. Correspondingly, the monomethyl metabolite AUC in mice treated with 750 mg m$^{-2}$ was 8 mM × minutes; but even at the MTD in patients (6,000 mg m$^{-2}$) the highest monomethyl AUC was 3.7 mM × minutes. Although the parent drug AUC in patients treated at the MTD (mean 559 mM × minutes) exceeded the parent drug AUC in mice treated with 750 mg m$^{-2}$ (142 mM × minutes), the monomethyl metabolite AUC in patients at the MTD was less than predicted, based on levels in mice. Therefore, although qualitative species similarities in CB10-277 metabolism in mice and patients were detected, clear quantitative differences exist. Not only do patients have a higher plasma clearance of the parent compound, they are less efficient in forming the monomethyl metabolite. Despite the monomethyl levels in patients being less than expected, based on the predictions from mice studies, they were more than double levels that have been shown to produce *in vitro* cytotoxicity with similar monomethyl triazenes (Gibson *et al.*, 1986; Gibson *et al.*, 1986*a*).

As with dacarbazine, nausea and vomiting were dose limiting. Except for the flushing sensation, which was short lived and self limiting, other toxicities were infrequent. Of particular interest was that no evidence of myelosuppression or liver function abnormalities were observed with administration of CB10-277 by short infusion. There were one complete and two partial responses in patients with metastatic melanoma. One patient with recurrent carcinoid also had a partial response. Additional evidence of antitumour activity (mixed responses, tumour necrosis at autopsy) occurred in patients with melanoma and sarcoma. Athough the responses occurred in patients who arguably might have responded to dacarbazine and occurred in subcutaneous, nodal or lung disease; the patients were not required to remain in the hospital for treatment nor return daily for 5 days. In addition, the nausea and vomiting, even in patients with the most severe symptoms, resolved by 24 h.

An additional Phase I study of CB10-277 using a 24 h continuous infusion was performed to investigate whether the severity of the acute nausea and vomiting could be reduced. Details of the 24 h continuous infusion study and further discussion of the rationale are given in the accompanying manuscript. The usefulness of CB10-277 in dacarbazine sensitive histologies can best be determined by a randomised comparison of the two drugs. The dose and schedule of CB10-277 recommended for future studies is given and discussed in the 24 h continuous infusion manuscript.

This work was supported by grants from the Cancer Research Campaign and Medical Research Council. The Phase I study was performed under the auspices of the Cancer Research Campaign Phase I Committee. Dr Foster is a recipient of an E.O.R.T.C./N.C.I.

Fellowship Award. We thank Mr Paul Davignon and Dr Omar Yoder for their assistance in obtaining formulated CB10-277. The authors wish to thank Miss Kathy Balmanno for her assistance in the preparation of this manuscript.

### References

AUDETTE, R.C.S., CONNORS, T.A., MANDEL, H.G., MERAI, K. & ROSS, W.C.J. (1973). Studies on the mechanism of action of the tumour inhibitory triazenes. *Biochem. Pharmacol.*, **22**, 1855-1864.

BERETTA, G., BONADONNA, G., BAJETTA, E., TANCINI, G., DE LENA, M., AZZARELLI, A. & UMBERTO, V. (1976). Combination chemotherapy with DTIC (NSC-45388) in advanced malignant melanoma, soft tissue sarcoma and Hodgkin's disease. *Cancer Treat. Rep.*, **60**, 205-211.

BONO, V.H. JR. (1976). Studies on the mechanism of action of DTIC (NSC-45388). *Cancer Treat. Rep.*, **60**, 141-148.

BRITISH INDUSTRIAL BIOLOGICAL RESEARCH ASSOCIATION (BIBRA) (1988) CB10-277. *Toxicologic Report.*

COLOMBO, T. & D'INCALCI, M. (1984). Comparison of the antitumor activity of DTIC and 1-p-(3,3-dimethyl-1-triazeno) benzoic acid potassium salt on murine transplantable tumors and their hematological toxicity. *Cancer Chemother. Pharmacol.*, **13**, 139-141.

COMIS, R.L. (1976). DTIC (NSC-45388) in malignant melanoma: a perspective. *Cancer Treat. Rep.*, **60**, 165-176.

CONNORS, T.A., GODDARD, P.M., MERAI, K., ROSS, W.C.J. & WILMAN, D.E.V. (1976). Tumour inhibitory triazenes: structural requirements for an active metabolite. *Biochem. Pharmacol.*, **25**, 241-246.

GESCHER, A., HICKMAN, J.A., SIMMONS, R.J., STEVENS, M.F.G. & VAUGHIN, K. (1981). Studies of the mode of action of antitumor triazenes and triazines. II. Investigation of the selective toxicity of 1-aryl-3,3-dimethyltriazenes. *Biochem. Pharmacol.*, **30**, 89-93.

GIBSON, N.W., HARTLEY, J., LA FRANCE, R.J. & VAUGHN, K. (1986*b*). Differential cytotoxicity and DNA-damaging effects produced in human cells of Mer+ and Mer− phenotypes by a series of alkyltriazenylimidazoles. *Carcinogenesis*, **7**, 259-265.

GIBSON, N.W., HARTLEY, J.A., LA FRANCE, R.J. & VAUGHN, K. (1986*a*). Differential cytotoxicity and DNA - damaging effects produced in human cells of the MER+ and MER− phenotypes by a series of 1-aryl-3 alkyltriazenes. *Cancer Res.*, **46**, 4999-5003.

GIRALDI, T., NISI, C., CONNORS, T.A. & GODDARD, P.M. (1977). Preparation and antitumour activity of 1-aryl 3,3-dimethyltriazene derivatives. *J. Med. Chem.*, **20**, 850-853.

HATHEWAY, G.J., HANSCH, C., KIM, K.H., MILSTEIN, S.R., SCHMIDT, C.L. & SMITH, R.N. (1978). Antitumor 1-(x-aryl) 3,3 dialkyl triazenes. 1. Quantitative structure - activity relationships vs L1210 leukemia in mice. *J. Med. Chem.*, **21**, 563-574.

JULLIARD, M. & VERNIN, G. (1981). Biological properties of antitumor triazenes. *Ind. Eng. Chem. Prod. Res. Dev.*, **20**, 287-296.

LOO, T.L. & LIN, Y.T. (1972). Preparation and antitumor activity of derivatives of 1-phenyl 3,3 dimethyltriazene. *J. Med. Chem.*, **15**, 201-203.

LOO, T.L., HOUSHOLDER, G.E., GERULATH, A.H., SAUNDERS, P.H. & FARQUHAR, D. (1976). Mechanism of action and pharmacology studies with DTIC (NSC-45388). *Cancer Treat. Rep.*, **60**, 149-152.

LUNN, J.M. & HARRIS, A.L. (1988). Cytotoxicity of 5-(3-methyl-1-triazeno)imidazole-4-carboxamide (MTIC) on Mer+, Mer+ Rem− and Mer− cell lines: differential potential by 3-acetamidobenzamide. *Br. J. Cancer*, **57**, 54-58.

MASTRANGELO, M.J., BAKER, A.R. & KATZ, H.R. (1985). Cutaneous melanoma. In *Cancer Principles and Practice of Oncology*, De Vita, V.T. Jr., Hellman, S. & Rosenberg, S.A. (eds). pp. 1371-1422. Lippincott, Philadelphia.

MORRISON, S.D. (1983). *In vivo* estimation of size of experimental tumors. *J. Natl Cancer Inst.*, **71**, 407-408.

POTTER, M. & ROBERTSON, C.L. (1960). Development of plasma cell neoplasms in BALB/c mice after intraperitoneal injection of paraffin-oil adjuvant heat killed *Staphylococcus* mixtures. *J. Natl Cancer Inst.*, **25**, 847-861.

ROSENOER, V.M., MITCHLEY, B.C.V., ROE, F.J.C. & CONNORS, T.A. (1966). Walker carcinosarcoma 256 in study of anticancer agents. I. Methods for simultaneous assessment of therapeutic value and toxicity. *Cancer Res.* (suppl) **26**, 937-941.

RUTTY, C.J., GRAHAM, M.A., ABEL, G., JUDSON, I.R. & GODDARD, P.M. (1986). Preclinical evaluation of 1-p-carboxy-3,3-dimethylphenyltriazene (CB10-277) an alternative to DTIC. *Br. J. Cancer*, **54**, 194.

SAVA, G., ZORZET, S., PERISSIN, L., GIRALDI, T. & LASSIANI, L. (1988). Effects of an inducer and an inhibitor of hepatic metabolism on the antitumor action of dimethyltriazenes. *Cancer Chemother. Pharmacol.*, **21**, 241-245.

SELBY, P.J., THOMAS, J.M., MONAGHAN, P., SLOANE, J. & PECKHAM, M.J. (1980). Human tumour xenografts established and serially transplanted in mice immunologically deprived by thymectomy, cytosine arabinoside and whole-body irradiation. *Br. J. Cancer*, **41**, 52-61.

SHEALY, Y.F., MONTGOMERY, J.A. & LASTER, W.R. Jr. (1962). Antitumor activity of triazenoimidazoles. *Biochem. Pharmacol.*, **11**, 674-676.

SHEALY, Y.F. & KRAUTH, C.A. (1966). Imidazoles II. 5 (or 4)-monosubstituted triazenoimidazole-4 (or 5)- carboxamides. *J. Med. Chem.*, **9**, 34-38.

TRANUM, B.L., DIXON, D., QUAGLIANA, J., NEIDHART, J., BALCERZAK, S.P., COSTANZI, J.H., FABIAN, C.J., NEILAN, B., MALONEY, T., O'BRYAN, R.M. & GROPPE, C. (1987). Lack of benefit of adjunctive chemotherapy in stage 1 malignant melanoma: A Southwest Oncology Group study. *Cancer Treat. Res.*, **71**, 643-644.

VAUGHN, K., TANG, L., LLANOS, G., HORTON, J.K., SIMMONDS, R.J., HICKMAN, J.A. & STEVENS, M.F.G. (1984). Studies of the mode of action of antitumor triazenes and triazines. 6. 1-aryl-3(hydroxymethyl- 3-methyltriazenes: synthesis, chemistry, and antitumor properties. *J. Med. Chem.*, **27**, 257-363.

VERONESI, V., ADAMUS, J., AUBERT, C., BAJETTA, E., BERETTA, G., BONADONNA, B., BUFALINO, R., CASCINELLI, N., COCONI, G., DURAND, J., DE MARSILLAC, J., IKONOPISOV, R.L., KISS, B., LEJEUNE, F., MACKIE, R., MADEJ, G., MULDER, H., MECHL, Z., MILTON, G.W., MORABITO, A., PETER, H., PRIARIO, J., PAUL, E., RUMKE, P., SERTOLI, R. & TOMIN, R. (1982). A randomized trial of adjuvant chemotherapy and immunotherapy in cutaneous melanoma. *N. Engl. J. Med.*, **307**, 913-916.

WHO Handbook for Reporting Results of Cancer Treatment: World Health Organization, *WHO Offset Publication* no. 48, Geneva, 1979.

WILMAN, D.E.V. & GODDARD, P.M. (1980). Tumour inhibitory triazenes. 2. Variation of antitumour activity within a homologous series. *J. Med. Chem.*, **23**, 1052-1054.